# Predicting seizures in pregnant women with epilepsy: Development and external validation of a prognostic model

**DOI:** 10.1371/journal.pmed.1002802

**Published:** 2019-05-13

**Authors:** John Allotey, Borja M. Fernandez-Felix, Javier Zamora, Ngawai Moss, Manny Bagary, Andrew Kelso, Rehan Khan, Joris A. M. van der Post, Ben W. Mol, Alexander M. Pirie, Dougall McCorry, Khalid S. Khan, Shakila Thangaratinam

**Affiliations:** 1 Barts Research Centre for Women’s Health, Barts and the London School of Medicine and Dentistry, Queen Mary University of London, London, United Kingdom; 2 Multidisciplinary Evidence Synthesis Hub, Queen Mary University of London, London, United Kingdom; 3 CIBER Epidemiology and Public Health, Madrid, Spain; 4 Clinical Biostatistics Unit, Hospital Ramón y Cajal, Madrid, Spain; 5 Patient and Public Involvement, Katie’s Team, Katherine Twining Network, Queen Mary University of London, London, United Kingdom; 6 Queen Elizabeth Hospital Birmingham, University Hospitals Birmingham NHS Foundation Trust, Birmingham, United Kingdom; 7 Department of Neurology, Royal London Hospital, Barts Health NHS Trust, London, United Kingdom; 8 Department of Obstetrics and Gynaecology, Royal London Hospital, Barts Health NHS Trust, London, United Kingdom; 9 Department of Obstetrics and Gynaecology, University of Amsterdam, Academic Medical Centre, Amsterdam, The Netherlands; 10 Monash University, Monash Medical Centre, Clayton, Victoria, Australia; 11 NHS Education for Scotland, Edinburgh, United Kingdom; King’s College London, UNITED KINGDOM

## Abstract

**Background:**

Seizures are the main cause of maternal death in women with epilepsy, but there are no tools for predicting seizures in pregnancy. We set out to develop and validate a prognostic model, using information collected during the antenatal booking visit, to predict seizure risk at any time in pregnancy and until 6 weeks postpartum in women with epilepsy on antiepileptic drugs.

**Methods and findings:**

We used datasets of a prospective cohort study (EMPiRE) of 527 pregnant women with epilepsy on medication recruited from 50 hospitals in the UK (4 November 2011–17 August 2014). The model development cohort comprised 399 women whose antiepileptic drug doses were adjusted based on clinical features only; the validation cohort comprised 128 women whose drug dose adjustments were informed by serum drug levels. The outcome was epileptic (non-eclamptic) seizure captured using diary records. We fitted the model using LASSO (least absolute shrinkage and selection operator) regression, and reported the performance using C-statistic (scale 0–1, values > 0.5 show discrimination) and calibration slope (scale 0–1, values near 1 show accuracy) with 95% confidence intervals (CIs). We determined the net benefit (a weighted sum of true positive and false positive classifications) of using the model, with various probability thresholds, to aid clinicians in making individualised decisions regarding, for example, referral to tertiary care, frequency and intensity of monitoring, and changes in antiepileptic medication. Seizures occurred in 183 women (46%, 183/399) in the model development cohort and in 57 women (45%, 57/128) in the validation cohort. The model included age at first seizure, baseline seizure classification, history of mental health disorder or learning difficulty, occurrence of tonic-clonic and non-tonic-clonic seizures in the 3 months before pregnancy, previous admission to hospital for seizures during pregnancy, and baseline dose of lamotrigine and levetiracetam. The C-statistic was 0.79 (95% CI 0.75, 0.84). On external validation, the model showed good performance (C-statistic 0.76, 95% CI 0.66, 0.85; calibration slope 0.93, 95% CI 0.44, 1.41) but with imprecise estimates. The EMPiRE model showed the highest net proportional benefit for predicted probability thresholds between 12% and 99%. Limitations of this study include the varied gestational ages of women at recruitment, retrospective patient recall of seizure history, potential variations in seizure classification, the small number of events in the validation cohort, and the clinical utility restricted to decision-making thresholds above 12%. The model findings may not be generalisable to low- and middle-income countries, or when information on all predictors is not available.

**Conclusions:**

The EMPiRE model showed good performance in predicting the risk of seizures in pregnant women with epilepsy who are prescribed antiepileptic drugs. Integration of the tool within the antenatal booking visit, deployed as a simple nomogram, can help to optimise care in women with epilepsy.

## Abstract 2

John Allotey and colleagues describe their model for predicting seizures in pregnant women with epilepsy.

## Author summary

**Why was this study done?:**

- Pregnant women with epilepsy are at increased risk of death and complications from seizures; their high-risk status during pregnancy and after childbirth is often not recognised.
- Knowledge of an individual’s risk of seizures could help healthcare professionals and pregnant women make decisions regarding management.
- To our knowledge, there are currently no models to predict risk of seizures in pregnant women with epilepsy on medication.

**What did the researchers do and find?:**

- We developed the EMPiRE model to predict the risk of seizures in pregnancy and up to 6 weeks after delivery in women with epilepsy on medication whose drug doses were managed based on clinical findings; we validated the model in a separate group of women whose dose management was based on drug levels in the blood.
- The model discriminated well between those with and without seizures, with good agreement between predicted and observed risks across both low- and high-risk women.
- The model is clinically useful for decision-making where the threshold of choice for seizure risk is between 12% and 99%.
- The model showed promising transportability to the validation cohort.

**What do these findings mean?:**

- The EMPiRE prediction model can be used by healthcare professionals to identify pregnant women at high risk of seizures and to plan early referral for specialist input; determine the need for close monitoring in pregnancy, labour, and after childbirth; and assess antiepileptic drug management.
- The performance of the model is unlikely to vary with the antiepileptic drug dose management strategy.

## Introduction

Women with epilepsy are 10 times more likely to die in pregnancy than those without the condition [1]—seizures are a common cause of death [2]. Despite warnings from consecutive reports of the Confidential Enquiry into Maternal Deaths (UK) on the failings in antenatal, intrapartum, and postnatal management of women with epilepsy, care of these women remains fragmented [3,4]. A lack of recognition of the women’s high-risk status by professionals in primary and in secondary care has been highlighted consistently as the main factor behind epilepsy-related maternal deaths [2,3,5]. Furthermore, up to 4 in 10 women discontinue their antiepileptic medication in pregnancy due to concerns about the effects of drugs on the fetus, thereby increasing their risk of seizures [6,7]. Many maternal deaths in women with epilepsy could be averted with timely specialist input [5]. Seizures in pregnancy also have a negative impact on daily living. For example, the loss of driving license following seizures affects employment, relationships, and quality of life [8–10].

Pregnant women with epilepsy at risk of seizures need a personalised management plan for antenatal, intrapartum, and postnatal care, which requires multidisciplinary input through joint obstetric neurology clinics; however, these clinics are not available in all healthcare centres [11]. Furthermore, women at high risk of seizures need close monitoring in labour, with adequate pain relief measures such as epidural analgesia, and use of long-acting benzodiazepines such as clobazam [11]. Current guidelines recommend the use of these measures in high-risk women [11]. But a lack of guidance on what constitutes high-risk pregnancy is one factor that has contributed to variations in the care of pregnant women with epilepsy [11].

Prediction of seizures based on a woman’s individual characteristics not only provides an accurate picture of the risks to inform decision-making, but also promotes effective communication between the multi-specialty teams caring for women with epilepsy. A tool for predicting seizure risk can empower women to make informed decisions on their antenatal and intrapartum care. Furthermore, awareness of one’s risk status may lower any anxiety arising from the unpredictable nature of seizures [12], and promote adherence to medication through risk-informed counselling [13].

To our knowledge, there are currently no models to predict seizure risk in pregnant women with epilepsy. Existing, small retrospective studies provide imprecise estimates of the performance of individual predictors, such as type of seizures and seizure status in pre-pregnancy [14–16]. We aimed to develop and externally validate a prognostic model to predict the risk of seizures in pregnant women with epilepsy on medication, until 6 weeks postpartum. We also planned to determine the net benefit of using the model at various threshold probabilities using decision curve analysis.

## Methods

We developed and validated the prognostic model for seizures in the prospective multicentre EMPiRE (AntiEpileptic drug Monitoring in PREgnancy) study, which recruited pregnant women with epilepsy on antiepileptic drugs at first antenatal visit from 50 maternity units in the UK between 4 November 2011 and 17 August 2014 [17]. The UK National Research Ethics Committee approved the EMPiRE study (11/WM/0164), written consent was obtained from participants, and the protocol can be accessed at https://www.journalslibrary.nihr.ac.uk/programmes/hta/095538#/. The research reported here did not require further review by an ethics committee. We reported our prognostic study in line with the TRIPOD (Transparent Reporting of a multivariable prediction model for Individual Prognosis Or Diagnosis) recommendations, and present our findings as a nomogram, a graphical representation of the model to calculate an individual’s risk of seizure [18–20] (S1 TRIPOD Checklist).

### Model development and validation cohorts

EMPiRE was a prospective study, and recruited pregnant women with epilepsy on lamotrigine, carbamazepine, phenytoin, or levetiracetam before 24 weeks’ gestation. Serum antiepileptic drug levels were assessed every month, but the women and clinicians were blinded to these levels. For women for whom drug levels remained stable, the blinding was maintained (non-randomised cohort) until delivery, and drug doses were adjusted based on clinical features, in line with national recommendations [17,21]. Women whose serum drug levels fell were randomly allocated either to a strategy of adjusting antiepileptic doses based on serum drug levels (after unblinding) or to a strategy of changing the drug doses based on only clinical features (blinding maintained). All participants were followed up until 6 weeks after delivery (S1 Appendix). The study is described in detail elsewhere [17]. For model development, we used the cohort of women who were managed without routine serum drug level monitoring (non-randomised and randomised women), which is in line with standard epilepsy care in the UK [17,21]. We validated the model in the separate cohort of women managed differently, with routine therapeutic drug monitoring, as practised in some countries such as the US, to determine if the model was transportable across varied healthcare practices [22].

### Candidate predictors

A multidisciplinary team of neurologists, obstetricians, and researchers selected the candidate predictors for further evaluation in the prognostic model, based on existing evidence and their relevance to clinical care [14,16,23–26]. From an initial list of 65 baseline variables, we selected the following candidate predictors: age at first seizure, history of learning difficulty or mental health disorder, baseline seizure classification (tonic-clonic, non-tonic-clonic, unspecified), history of seizure in the 3 months before pregnancy (tonic-clonic, non-tonic-clonic), number of seizures between start of pregnancy and baseline visit, type of antiepileptic drug taken at baseline, dose of antiepileptic drug taken at baseline, gestational age at baseline, and hospital admission for seizures in a previous pregnancy. All continuous predictors were assumed to be linearly associated with the outcome.

### Outcome

Our main outcome was the occurrence of tonic-clonic (convulsive) or non-tonic-clonic (non-convulsive) seizure [27]. Participants prospectively recorded their epileptic seizures, if any, in purpose-built seizure diaries. To avoid overfitting of multivariable models, the rule of thumb is to ensure that there are 10 events for each predictor variable that was considered for inclusion in the model [28]; we worked within this rule limitation.

We assessed the predictive performance of the model using measures of discrimination (C-statistic) and accuracy (calibration slope). The C-statistic represents the ability of the model to discriminate between those who do and do not experience seizures; a value of 1 indicates perfect discrimination, and a value of 0.5 indicates no discrimination beyond chance [29]. Models are considered to have a good performance when the C-statistic exceeds 0.7 [30]. Calibration refers to agreement between the predicted and observed risk of seizure for all groups of predicted probabilities. A well-calibrated model will have a calibration slope of 1, and all groups will fit close to this line.

### Statistical analysis

#### Model development

Missing values were imputed using 10-fold multiple imputation by chained equations. Within each imputed dataset we developed a multivariable logistic regression model using the LASSO (least absolute shrinkage and selection operator) method, which simultaneously selects the variables and penalises the model coefficients for over-optimism. We selected the lambda parameter that minimised expected model deviance. Final coefficients were combined across imputed datasets using Rubin’s rule [31,32]. Confidence intervals for model coefficients were obtained by bootstrap sampling. Bootstrap validation was carried out to adjust the performance of the model for optimism. We repeated the entire modelling process on 100 bootstrap samples drawn from each of the 10 imputed datasets. We did not consider the number of tonic-clonic seizures between the diagnosis of pregnancy and baseline visit as a variable, as it was highly correlated with the predictor variable of tonic-clonic seizure history in the 3 months before pregnancy.

We assessed the overall discriminatory ability of the model using the C-statistic (summarised as the area under receiver operating characteristic curve [AUC ROC]) with 95% confidence interval (CI). Model calibration was visually assessed with a calibration plot representing deciles of predicted probability of seizure against the observed rate in each risk group. The calibration slope was also calculated, which is the slope of the regression line fitted between predicted and observed risk probabilities on the logit scale, with 1 being the ideal value.

#### External validation

We externally validated the final model in a separate cohort of women whose drug levels were routinely monitored (therapeutic drug monitoring) to plan drug dose adjustments. We report the predictive performance in this cohort using the same measures of discrimination and calibration as used in the model development cohort [33]. We visually assessed the model’s calibration by plotting quartiles of predicted probability of seizure against the observed rate in each quartile, and estimated the calibration slope [34,35].

#### Sensitivity analysis

We undertook sensitivity analysis by combining all available data in the development and validation cohorts to determine if there was any change in model performance. We evaluated antiepileptic drug dose monitoring strategy (therapeutic drug monitoring versus clinical features monitoring) as a predictor while developing the combined model.

#### Decision curve analysis

We performed decision curve analysis to assess the clinical value of the model. A decision rule should take into consideration the identification of women likely to have seizures because of the significant consequences (maternal death, accidents, loss of driving license), and the avoidance of unnecessary interventions leading to adverse impact on mother or baby. We determined the net benefit of the model across a wide range of threshold probabilities, instead of simply classifying all pregnant women with epilepsy as predicted to have seizures or classifying no women as predicted to have seizures [36]. We represent the net benefit as a function of the decision threshold in a decision curve plot.

The choice of thresholds will vary according to the planned intervention, the preference of the clinician, and the preference of the mother. For example, if the intervention involves referral of a pregnant woman with epilepsy to a tertiary unit for antenatal and intrapartum care, at a low threshold, false negatives are minimised at the expense of unnecessary referrals to tertiary care. At a high threshold, fewer women are referred, but women who are likely to benefit may be denied access to tertiary care. We expect an intermediate range of thresholds to be clinically acceptable. But if the planned intervention is to increase the dose and number of antiepileptic drugs—which can have adverse side effects on the mother and increase the risk of long-term neurodevelopmental problems in the child—clinicians may choose a higher threshold than what was chosen for tertiary referral.

#### Nomogram

We also developed a simple, easy-to-use nomogram to calculate the predicted probability of seizures in pregnant women at the time of antenatal booking.

The analyses were performed using Stata software version 15.1 and R software version 3.3.2 [37,38].

## Results

The EMPiRE study recruited 560 pregnant women. The model development cohort included 399 women; the validation cohort included 128 women (S1 Appendix).

### Characteristics of the women

The average gestational age at baseline was 16.6 weeks (SD 4.0) in the development cohort and 14.9 weeks (SD 4.4) in the validation cohort (Table 1). The mean age at first seizure was similar in both cohorts, at 16 years, and 10%–15% of women had a learning difficulty or mental health disorder. A similar proportion of women in the development and validation cohorts were previously diagnosed to have tonic-clonic seizures (development, 39%; validation, 36%). Overall, 46% (182/399) of women in the development cohort and 39% (50/128) in the validation cohort had experienced seizures in the 3 months prior to pregnancy. Lamotrigine was the commonest antiepileptic drug prescribed in both cohorts; more than half of the women took lamotrigine in the development (226/399, 57%) and validation cohorts (80/128, 63%).

**Table 1:** Details of women’s characteristics in the development and validation cohorts of the EMPiRE prediction model and the proportion with missing data.

Characteristic	Development cohort (*n* = 399)		Validation cohort (*n* = 128)	
	Mean (SD) or *n* (%)	Number with missing data, *n* (%)	Mean (SD) or *n* (%)	Number with missing data, *n* (%)
Gestational age at baseline (weeks)	16.6 (4.0)	0	14.9 (4.4)	0
History of learning difficulty or mental illness	50 (13%)	1 (0.3%)	20 (16%)	0
Age at first seizure (years)	16.5 (7.4)	5 (1.3%)	16.8 (7.8)	0
≤10 years	70 (17.8%)		28 (21.9%)	
11–20 years	215 (54.6%)		59 (46.1%)	
21–30 years	94 (23.9%)		35 (27.3%)	
31–40 years	15 (3.8%)		6 (4.7%)	
Admission to hospital for seizures in previous pregnancy	28 (7.0%)	22 (5.5%)	14 (10.9%)	7 (5.5%)
Seizure classification at baseline				
Tonic-clonic	155 (39%)	0	46 (36%)	0
Non-tonic-clonic	232 (58%)	0	78 (61%)	0
Unspecified	12 (3%)	0	4 (3%)	0
Seizure in the 3 months before pregnancy	182 (46%)		50 (39%)	
Tonic-clonic	52 (13%)	83 (20.8%)	12 (9%)	32 (25.0%)
Non-tonic-clonic	130 (33%)	0	38 (30%)	0
Number of seizures in pregnancy prior to the baseline visit				
Tonic-clonic	0.7 (2.8)	82 (20.6%)	0.4 (2.1)	31 (24.2%)
Non-tonic-clonic	11.6 (108.4)	0	18.1 (83.4)	0
Antiepileptic drug intake at baseline				
Carbamazepine	74 (19%)	0	16 (13%)	0
Lamotrigine	200 (50%)	0	66 (52%)	0
Levetiracetam	99 (25%)	0	31 (24%)	0
Phenytoin	0	0	1 (1%)	0
Lamotrigine and carbamazepine	1 (0.3%)	0	—	0
Lamotrigine and levetiracetam	25 (6%)	0	14 (11%)	0
Baseline dose of antiepileptic drugs (mg/day)				
Carbamazepine	706.0 (348.5)	0	612.5 (346.2)	0
Lamotrigine	272.1 (155.6)	0	269.4 (160.6)	0
Levetiracetam	1,641.3 (886.8)	0	1,533.3 (760.5)	0
Phenytoin	0	0	200 (—)	0

### Model performance

**Fig 1:**
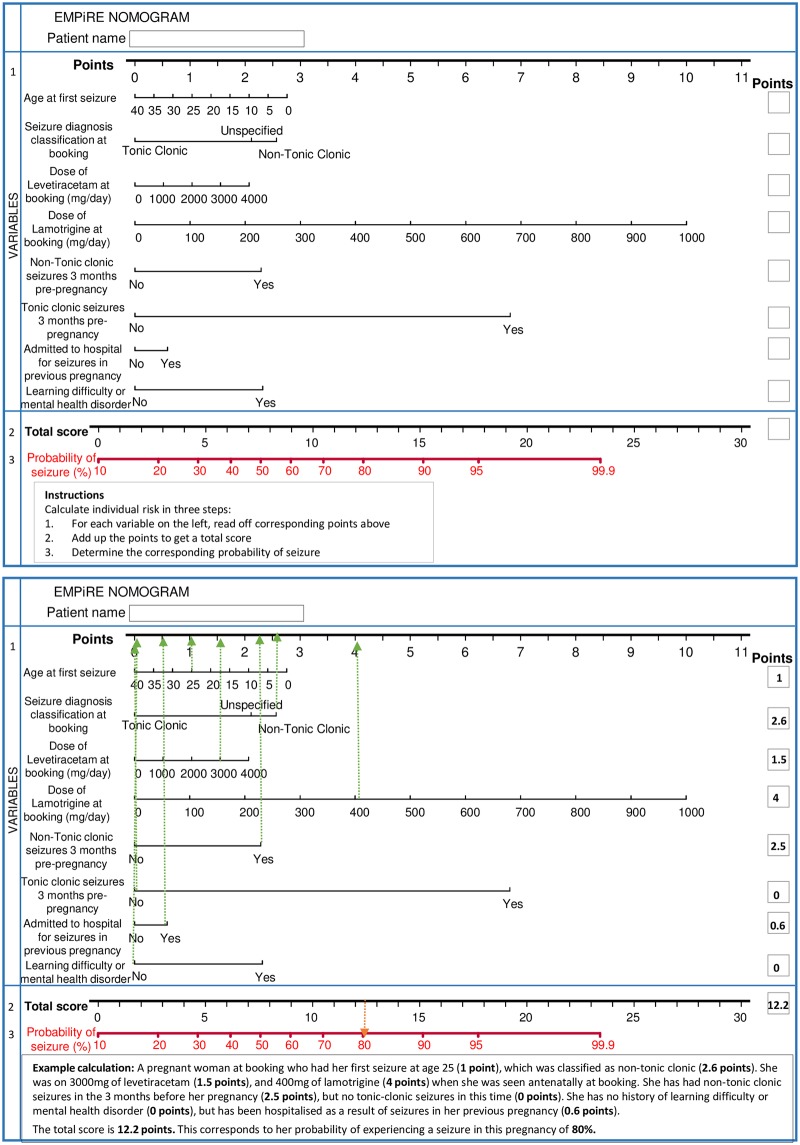
EMPiRE nomogram for predicting the risk of seizures at antenatal booking in pregnant women with epilepsy on antiepileptic drugs: A worked example.

Overall, 46% (183/399) of women in the development cohort experienced 1 or more seizures at any time from baseline until 6 weeks after delivery. Tonic-clonic seizures accounted for half of all seizures (90/183, 49%). Eight predictors were significantly associated with seizures and were included in the final multivariable model: age at first seizure, history of mental health disorder or learning difficulty, baseline seizure classification (tonic-clonic, non-tonic-clonic, unspecified), hospital admission for seizures in a previous pregnancy, tonic-clonic seizure in the 3 months before pregnancy, non-tonic-clonic seizure in the 3 months before pregnancy, baseline dose of lamotrigine, and baseline dose of levetiracetam (Table 2). The model is presented as a graphical calculator (nomogram) in Fig 1.

**Table 2:** Multivariable LASSO logistic regression of seizure risk prediction in pregnant women with epilepsy.

Candidate predictor	Multivariable analysis after MI (*n* = 399)	
	OR	Bootstrap 95% CI
Age at first seizure (years)	0.98	0.97, 0.99
History of learning difficulty or mental illness	1.96	1.68, 2.89
Seizure classification at baseline (ref. tonic-clonic)		
Non-tonic-clonic	2.11	1.88, 2.62
Unspecified	1.85	1.64, 4.30
Tonic-clonic seizure in the 3 months prior to pregnancy	7.20	6.63, 11.93
Non-tonic-clonic seizure in the 3 months prior to pregnancy	1.94	1.71, 2.38
Baseline dose of lamotrigine (×100 mg/day)	1.34	1.30, 1.44
Baseline dose of levetiracetam (×100 mg/day)	1.02	1.01, 1.03
Admitted to hospital for seizures in previous pregnancy	1.19	1.08, 1.92
Baseline dose of carbamazepine (×100 mg/day)	—	—
Number of non-tonic-clonic seizures since the start of pregnancy	—	—
Gestational age at baseline (weeks)	—	—

95% CI: Bootstrap limits of the confidence interval obtained from percentiles 2.5 and 97.5. Missing values were imputed using 10-fold MI by chained equations (step 1). We fitted a regression model using the LASSO strategy in each of the 10 imputed datasets (step 2). We averaged model coefficients using Rubin’s rule to get the final model coefficients (step 3). To obtain non-parametric 95% confidence intervals for model coefficients, we repeated the previous step 2 and step 3 on 1,000 bootstrap samples. Limits of the 95% confidence interval for each coefficient were the 2.5th and 97.5th percentiles of their distribution.

MI, multiple imputation; OR, odds ratio.

The equation of the EMPiRE prediction model for risk of seizures during pregnancy and until 6 weeks after delivery in women with epilepsy on antiepileptic drugs was as follows:

probability(seizure)=exp(Y)/(1+exp(Y))

where *Y* = −1.39 + (−0.02 * age at first seizure) + 0.61 [unspecified seizures] + 0.75 [non-tonic-clonic seizures] + (0.02 * dose of levetiracetam/100) + (0.29 * dose of lamotrigine/100) + 0.66 [non-tonic-clonic seizures in the 3 months before pregnancy] + 1.97 [tonic-clonic seizures in the 3 months before pregnancy] + 0.67 [learning difficulty or mental health disorder] + 0.17 [admitted to hospital for seizures during previous pregnancy].

All variables were coded as binary (1 when present and 0 when absent) except for age at first seizure (years), dose of lamotrigine (mg/day), and dose of levetiracetam (mg/day).

**Fig 2:**
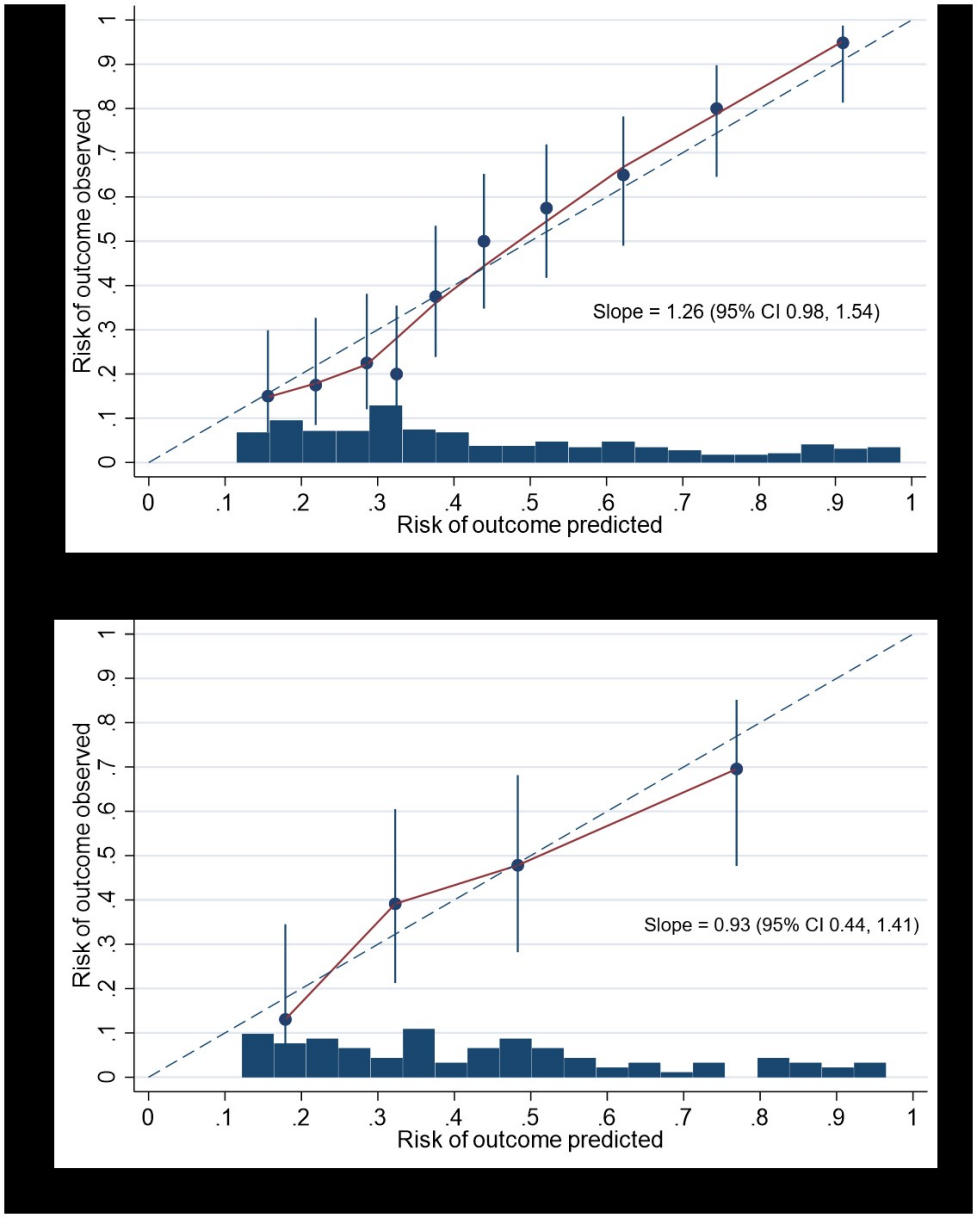
Calibration of the EMPiRE prediction model by comparing observed versus predicted risk of seizures in pregnant women on antiepileptic drugs, with a frequency histogram. Top panel = development cohort; bottom panel = validation cohort.

The apparent C-statistic for the model was 0.80 (95% CI 0.76, 0.85). After bootstrap adjustment for optimism, the final prediction model had a C-statistic of 0.79 (95% CI 0.75, 0.84) to discriminate between women with and without seizures (Table 3). The optimism-adjusted calibration plot (Fig 2) showed mostly good agreement between the predicted and observed risks, and the calibration slope was 1.26 (95% CI 0.98, 1.54).

**Table 3:** EMPiRE model performance.

Performance measure	Development cohort (*n* = 399)	Validation cohort (*n* = 128)
C-statistic	0.79 (95% CI 0.75, 0.84)	0.76 (95% CI 0.66, 0.85)
Calibration slope	1.26 (95% CI 0.98, 1.54)	0.93 (95% CI 0.44, 1.41)

Our sensitivity analysis, which combined all available data (*n* = 527), resulted in a model with the same predictors and similar coefficients as the EMPiRE model developed using only the development cohort. The antiepileptic drug monitoring strategy was not found to be a significant predictor of seizures and was therefore not selected in the combined model. The C-statistic and calibration slope of the combined model were 0.78 (95% CI 0.74, 0.82) and 1.22 (95% CI 0.44, 1.46), respectively (S2 Appendix).

### External validation and predictive performance

In the external validation cohort, 45% (57/128) of women experienced seizures at any time from baseline until 6 weeks after delivery; tonic-clonic seizures were reported in 39% (22/57) of women who had seizures. The final model showed good discrimination when externally validated, with a C-statistic of 0.76 (95% CI 0.66, 0.85). The model showed mostly good agreement between the predicted and observed risks, with a calibration slope of 0.93 (95% CI 0.44, 1.41) (Fig 2).

### Net benefit of model use

**Fig 3:**
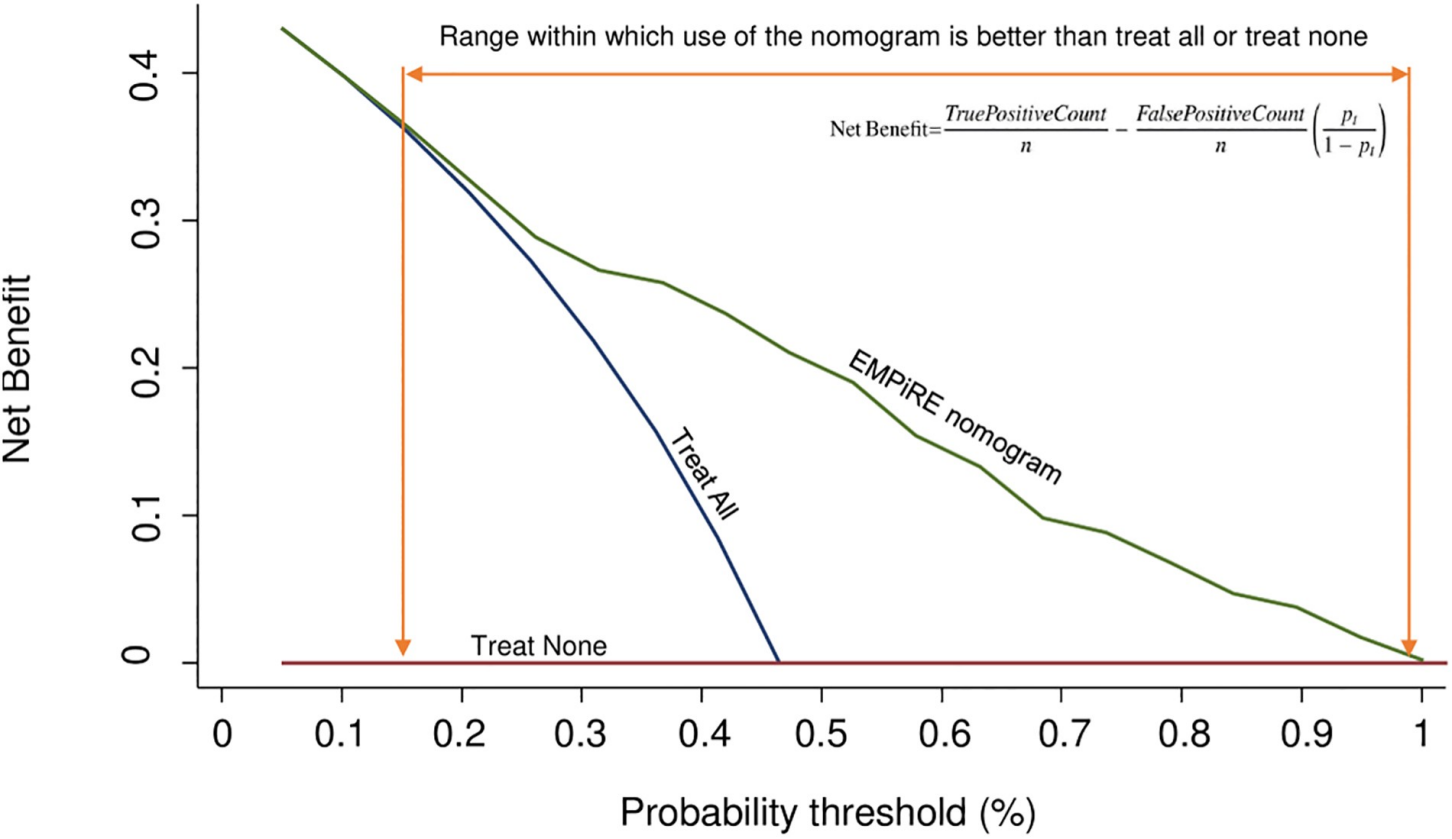
Decision curve analysis using the EMPiRE seizure risk prediction model. Red line (treat none) = net benefit when we assume that no pregnant woman with epilepsy will have the outcome (seizure in pregnancy); blue line (treat all) = net benefit when we assume that all pregnant women with epilepsy will have the outcome; green line (EMPiRE nomogram) = net benefit when we manage pregnant women with epilepsy according to the predicted risk of the outcome (seizure in pregnancy) estimated by the EMPiRE model. The preferred strategy is the one with the highest net benefit at any given threshold.

In our decision curve analysis (Fig 3), the curve for the EMPiRE model showed positive net benefit for predicted probability thresholds between 12% and 99% compared to managing pregnant women with epilepsy as if they will all have seizures or managing them as if none of them will have seizures (i.e., treat-all or treat-none strategies). Table 4 provides estimates of the net benefit of using the model for various probability thresholds. For low thresholds, below 12%, there was no difference between using the EMPiRE model and treating women as if they will all have seizures.

**Table 4:** Net benefit of using the EMPiRE prediction model compared to managing women with epilepsy assuming all of them will have seizures in pregnancy or the postpartum period.

Threshold probability	Net benefit		Advantage of using the model	
	Treat all women	EMPiRE model	Difference in net benefit	Reduction in number who do not need the intervention per 100 women
0.05	0.430	0.430	0	0
0.1	0.398	0.398	0	0
0.15	0.363	0.368	0.003	2
0.2	0.323	0.329	0.008	3
0.25	0.278	0.299	0.018	6
0.3	0.227	0.276	0.049	11
0.35	0.167	0.266	0.100	19
0.4	0.098	0.248	0.150	22
0.45	0.016	0.213	0.207	25
0.5	−0.083	0.193	0.286	29
0.55	−0.203	0.166	0.372	30
0.6	−0.353	0.164	0.503	34
0.65	−0.547	0.120	0.663	36
0.7	−0.805	0.111	0.911	39
0.75	−1.165	0.088	1.253	42
0.8	−1.707	0.073	1.774	44
0.85	−2.609	0.057	2.668	47
0.9	−4.414	0.030	4.454	49
0.95	−9.827	0.023	9.852	52
0.99	−53.135	0.000	53.135	54

## Discussion

### Summary of the findings

The EMPiRE model performs well in predicting the risk of seizures at the time of antenatal booking in pregnant women with epilepsy who are prescribed antiepileptic medication. The model incorporates routinely available characteristics that are easy to measure, such as age at first seizure, type of seizures, seizures in the 3 months before pregnancy, mental health, admission to hospital for seizures during a previous pregnancy, and dose of antiepileptic drugs. The model is clinically useful over a range of threshold probabilities, and is relevant to general practitioners, epilepsy specialists, obstetricians, and midwives in identifying high-risk women. The model shows potential for transportability across risk groups to settings where routine therapeutic drug monitoring is undertaken, but the findings should be interpreted with caution due to the small number of events in the validation sample. Our simple nomogram is designed to facilitate the model’s use in clinical practice.

### Strengths and limitations

To our knowledge, ours is the only clinical prognostic model to predict seizures in pregnant women with epilepsy. We developed the model using data from a prospective, high-quality, multicentre study. We evaluated predictors that were clinically relevant and routinely available to healthcare professionals, so that the model can be easily applied in clinical practice. Missing values of predictors were dealt with by multiple imputation, thereby avoiding loss of useful information [39,40]. We developed the model to predict seizures not only in pregnancy, but up to 6 weeks after delivery, a period with increased risks to the mother and baby [11,41]. We adjusted for optimism and addressed issues around overfitting in the model. In addition to providing the model as an easy-to-use nomogram, we provided information on its clinical use at various threshold probabilities for decision-making [35]. The model includes clinical variables that are easily accessible at the time of booking for incorporation into an app or integration into computer systems within healthcare services. A convenient and easy-to-use nomogram of the EMPiRE model allows for immediate use of the model to predict the risk of seizures without the need to remember the formulae behind it.

Our model development and validation approach took into account the significant variations in the management of antiepileptic drug dosages in pregnancy to prevent seizures [42]. While the American Academy of Neurology recommends routine serum therapeutic drug monitoring, with dosage increased if the serum drug level falls [22], the UK National Institute for Health and Care Excellence, Royal College of Obstetricians and Gynaecologists, and Scottish Intercollegiate Guidelines Network guidelines do not recommend routine drug monitoring but drug dose adjustments based mainly on clinical features [11,21,43]. In this paper, we developed and internally validated the model in women whose drug dose was managed based on clinical features to determine the accuracy and reproducibility of the model. Through our external validation, we assessed the transportability of the model to women managed using a different management strategy (therapeutic drug monitoring), which appears promising [44]. Furthermore, when we developed the combined model by using all available data (including women routinely and not routinely monitored for drug levels) in our sensitivity analysis, we did not observe any differences either in the number and type of predictors or in the model’s performance compared to the model developed using only women in the development cohort. The antiepileptic drug dose monitoring strategy was not identified to be a significant predictor in the combined model, implying that the model is generalisable irrespective of the strategy.

There are some limitations to this study. The cohorts consisted of women recruited with pre-specified criteria, which may limit the use of the model in all women [19,45]. We did not include women on sodium valproate; this is consistent with current recommendations against valproate use in pregnancy, due to the increased risk of birth defects and neurodevelopmental disorders [46]. The model can only be used in women managed on phenytoin, lamotrigine, levetiracetam, or carbamazepine and when information is available on all predictors in the context of care in a high-income setting. This limits its transportability to low- and middle-income countries with resource constraints and non-availability of these drugs [47]. Women were recruited at varied gestational ages. However, we evaluated gestational age as a predictor, and it was not selected by the modelling strategy in the final model. The model included history of seizures in the 3 months before pregnancy obtained through retrospective recall, with resultant bias. We consider this to reflect the real life scenario, where women who do not receive pre-pregnancy specialist epilepsy care often do not maintain a prospective seizure diary prior to seeing an epilepsy specialist in pregnancy. It is possible that a different predictor such as history of seizures in the 9 months before pregnancy instead of the 3-month history in our model may have improved its performance [48,49]. We only included clinical predictors routinely available at the time of antenatal booking, and did not evaluate other tests such as electroencephalogram (EEG) or MRI, or risk factors such as history of nocturnal or prolonged seizures, which may be available in specialist epilepsy care. We could not assess any changes in antiepileptic medication before conception because this information was not routinely recorded at antenatal booking, and was not collected in the EMPiRE trial. These additional variables may have improved the performance of the model. Due to the small sample size and the small number of events in the validation cohort (<100), we were limited in our interpretation of the transportability of the model [50].

### Comparison to existing evidence

To our knowledge, 2 other prediction models exist for seizures, both involving non-pregnant individuals: seizure prediction in children and adults who have recently stopped their antiepileptic drugs, reported using individual participant data (IPD) meta-analysis, and prediction of subsequent seizures after a single seizure in individuals without clear indication to commence treatment (MESS study) [51,52]. Some predictors in the IPD meta-analysis model such as the age at onset of epilepsy were also present in our model. It is not appropriate or feasible to apply other variables, such as seizure-free interval before antiepileptic drug withdrawal and epileptiform abnormality on EEG, to the pregnant population [51]. The performance of our EMPiRE model was better than that of the IPD meta-analysis model (C-statistic 0.65) [51]. The MESS study, which used split sample validation, did not report the performance of the model with currently recommended measures such as C-statistic and calibration slope, and hence we are unable to compare that model with the EMPiRE model.

Other individual studies such as the EURAP (European and International Registry of Antiepileptic Drugs and Pregnancy) have reported on the association between maternal risk factors and seizures in pregnancy—none provided multivariable prognostic models [14,16]. Compared to a third of pregnant women with epilepsy on medication developing seizures in the EURAP study, 46% of women in the EMPiRE cohorts experienced seizures in pregnancy or until 6 weeks after delivery [14,23]. This difference could be attributable to the known increase in seizures that occurs after delivery in new mothers, as EURAP did not include postnatal mothers or prospective seizure diaries [41]. Inclusion of a selective group of women in the EMPiRE study may also have contributed to the difference. Similarly to the EURAP study, our final model identified lamotrigine dose to be a predictor of seizures [14]. Another small retrospective study identified pre-pregnancy seizure status to be the main predictor of seizures in pregnancy [16], which was also the strongest predictor of seizures in our model.

### Relevance to clinical care

Currently pregnant women with epilepsy are managed by varied healthcare professionals such as general practitioners, obstetricians and midwives, and epilepsy specialists. There is no clear pathway for multidisciplinary communication. Joint obstetric neurology clinics are not available in half of the maternity units in UK, a major hindrance for integration of epilepsy care within antenatal care [53]. The first step towards achieving integrated care is effective risk communication of the mother’s seizure status. Such a risk-based approach using quantified risk estimates can help to avoid maternal deaths such as those reported in the MBRRACE-UK (Mothers and Babies: Reducing Risk through Audits and Confidential Enquiries across the UK) report [5], where women with epilepsy were never treated by an epilepsy specialist in pregnancy, and were left unmonitored during their hospital admissions without specialist input [5].

We refrained from recommending specific decision thresholds for various interventions as these are likely to vary with the potential adverse effects and costs of the planned intervention. For example, primary care clinicians may consider a 20% cutoff, a level of risk associated with driving restrictions, to be appropriate to make decisions on early referral to tertiary units with joint obstetric neurology clinics. However, secondary care clinicians may choose a higher threshold when the intervention involves frequent antenatal monitoring (weekly or fortnightly), intrapartum use of invasive interventions for pain relief such as epidural and other medications (such as clobazam, which carries a risk of neonatal respiratory depression), or close monitoring in the postnatal period. The choice of threshold in a clinical setting is also likely to vary depending on the epilepsy syndrome and seizure types. For example, the intervention threshold may be lower for patients who tend to experience convulsive seizures than for those who experience absence seizures.

Women’s choice of thresholds may depend on the additional time and resources required (for example, long-distance travel to access tertiary care) and the perceived risks to themselves and their babies from the various interventions. If the ability to drive is crucial to the mother for her job and other responsibilities, after discussion with clinicians, she may opt for a lower threshold for interventions in secondary care. But if minimising the risk of long-term adverse offspring neurodevelopmental outcomes is valued more by the mother than minimising the risk of seizures, she may choose a higher threshold for increasing the dose and number of antiepileptic drugs. Our decision curve analysis shows that the model is useful across a wide range of threshold probabilities.

Use of the model in clinical practice should be complementary to individualised advice on safety, risk assessment, drug adherence, and triggers for seizures. Awareness of seizure risk can minimise non-adherence to medication in pregnancy, one of the major factors behind seizure deterioration in pregnancy [6,7,13]. Women predicted to have a low risk of seizure by the model should be informed that their risk status is subject to adherence to their antiepileptic medication. The EMPiRE model does not identify women below 12% risk. Women and clinicians should be aware of this limitation if the probability threshold to make decisions on eligibility for home or water birth is below this threshold.

### Relevance to research

The effect of the addition of other markers, such as EEG findings or historical MRI brain imaging reports, on the performance of the model needs further evaluation. There is a need for multiple external validations across different settings and populations to fully appreciate the transportability of the model [54]. The impact of using the EMPiRE model in clinical practice needs to be evaluated through cluster-randomised trials, to assess whether it helps improve outcomes such as the seizure-free period or quality of life of these women. While the tool is expected to improve women’s knowledge of their risk status for seizures in pregnancy, the effect of the EMPiRE model on women’s anxiety levels is not known and needs to be assessed. Further studies are needed to assess the acceptability of the tool to women with epilepsy and to healthcare providers, their preferred thresholds of choice, and the cost utilities of consequences of decisions for various false positive and false negative cases.

### Conclusions

The EMPiRE nomogram is a simple 8-item prediction tool to calculate the individualised risk of seizures at antenatal booking in pregnant women with epilepsy on antiepileptic drugs. The estimates can help guide individually tailored choices made by patients and clinicians, which may influence the intensity of monitoring in pregnancy and after delivery, place of care, and antiepileptic drug dose adjustment strategy. The model is not clinically useful for decision-making at very low thresholds.

## Supporting information

S1 Appendix Flow diagram of EMPiRE trial participants contributing to model development and external validation. (TIF)

S2 Appendix Result of sensitivity analysis combining all available data. (DOCX)

S1 TRIPOD Checklist (DOCX)

## Abbreviations

EEG: electroencephalogram
IPD: individual participant data
