# Supplementary figures and images for: Predicting seizures in pregnant women with epilepsy: Development and external validation of a prognostic model
Source: PLoS Med. 2019 May 13;16(5):e1002802. doi: 10.1371/journal.pmed.1002802 (PMC6513048; doi:10.1371/journal.pmed.1002802)

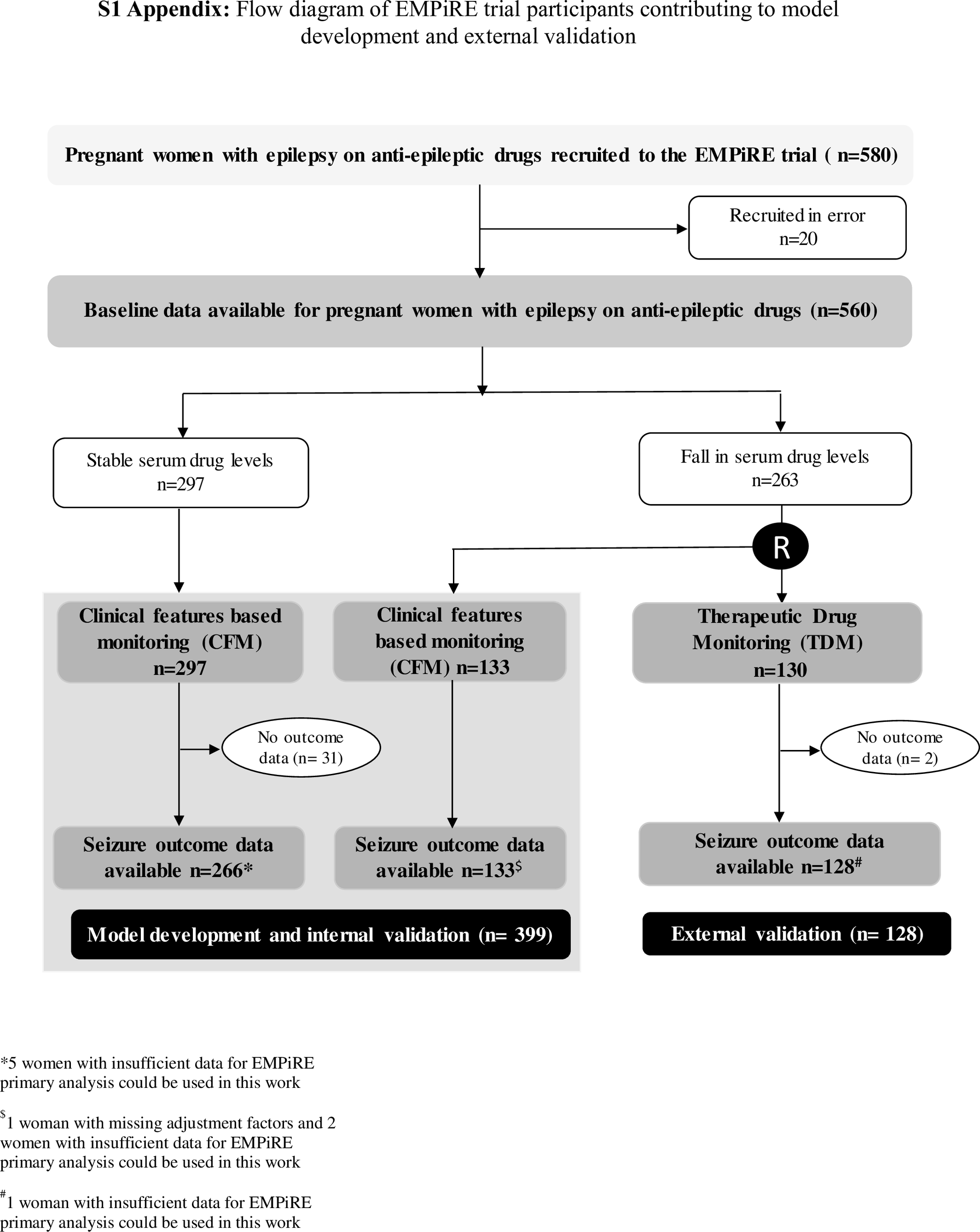

Supplement: S1 Appendix — (TIF) [file pmed.1002802.s001.tif]
