# Supplementary material for: Predicting seizures in pregnant women with epilepsy: Development and external validation of a prognostic model
Source: PLoS Med. 2019 May 13;16(5):e1002802. doi: 10.1371/journal.pmed.1002802 (PMC6513048; doi:10.1371/journal.pmed.1002802)
Supplement: S2 Appendix — (DOCX) [file pmed.1002802.s002.docx]

**S2 Appendix: Result of sensitivity analysis combining all available data**

1. **LASSO Regression Model**

| Variables | n = 527 (Events = 240) | |
| --- | --- | --- |
|  | Coefficient | Odds Ratio |
| Intercept | -1.513 |  |
| Age at first seizure | -0.015 | 0.99 |
| History of learning or mental difficulty | 0.325 | 1.38 |
| Seizure classification at baseline (Ref. TCS) |  |  |
| Non-Tonic clonic | 0.884 | 2.42 |
| Unspecified | 0.160 | 1.17 |
| Tonic Clonic 3 months prior to pregnancy | 1.963 | 7.12 |
| Non-Tonic Clonic 3 months prior to pregnancy | 0.738 | 2.09 |
| Baseline dose of Lamotrigine (x100mg) | 0.244 | 1.28 |
| Baseline dose of Levetiracetam (x100mg) | 0.018 | 1.02 |
| Admitted to hospital for seizures in previous pregnancy | 0.360 | 1.43 |
| Baseline dose of Carbamizepine (x100mg) | - | - |
| Number of non-tonic clonic seizure since the start of pregnancy | - | - |
| Gestational age at baseline | - | - |
| Cohort | - | - |
| EMPiRE model performance |  | |
| AUC apparent | 0.79 (0.76; 0.83) | |
| AUC adjusted | 0.78 (0.74 ; 0.82) | |
| Slope | 1.22 (0.99; 1.46) | |

1. **CALIBRATION PLOT**

Development Cohort

1. **DECISION CURVE ANALYSIS**
